# Authors’ Response to Peer Reviews of “Influence of Mass Media on Italian Web Users During the COVID-19 Pandemic: Infodemiological Analysis”

**DOI:** 10.2196/34138

**Published:** 2021-10-18

**Authors:** Alessandro Rovetta, Lucia Castaldo

**Affiliations:** 1 Research and Disclosure Division Mensana srls Brescia Italy

**Keywords:** COVID-19, Google Trends, infodemiology, infoveillance, infodemic, media coverage, mass media influence, mass media, social media

*This is the authors’ response to peer-review reports for “Influence of Mass Media on Italian Web Users During the COVID-19 Pandemic: Infodemiological Analysis.”*

## Round 1 Review

### Reviewer E

1. Dear Reviewer [1], thanks for the warning. The text [2] has been double-checked. If there are any further inaccuracies, the manuscript will be sent to a proofreading service.

2. Dear Reviewer, the *Introduction* section has been rewritten and enriched following your instructions. Thank you so much for the valuable advice. Changed text is highlighted in blue in the new file.

3. Dear Reviewer, thank you for raising this important issue. The answer given was the following: “The internet represents a fast, user-friendly means to seek health-related information. Especially during a pandemic or any major health crisis, the need for quality web information is more pressing than ever: fear, anxiety, stress, and confusion due to the overabundance of often conflicting or dramatic news increases the consultation of web sources to seek remedies or reassurance.”

4. Dear Reviewer, once again, thanks for the valuable comment. The answer to this question is too long to be reported here. However, we created the *Countermeasures to the Infodemic* subsection to discuss this point.

5. Dear Reviewer, thanks for the help and references provided. The use of Google Trends has been addressed in the subsection *Countermeasures to the Infodemic*.

6. Dear Reviewer, we agree that this subsection is too limited to fully represent the influence of the infodemic information reported by the mass media on users. However, we think it is important to point out that infodemic videos and articles have led to a great media clamor and a very high number of views. Indeed, these have been shared by the main information channels in Italy, thus reaching a vast audience. Since conspiracy theorists are subject to confirmation bias [3], absolute visibility is as relevant as relative visibility. For this reason, we think it important that a peer-reviewed source denounces this dangerous phenomenon. In this regard, we changed the name of the research question from “What was the role of the Italian mass media in the COVID-19 infodemic?” to “Among the contents proposed by mass media channels, what was the interest aroused by the infodemic news?” As for the definition of infodemic news, we drew on the infodemic scale (which has now been appropriately explained in the *Methods* section).

7. Dear Reviewer, thank you for noting this. We agree with you. We have removed R4 from the manuscript. To mention the matter, we wrote: “Alongside this, our analysis can provide further evidence on the reliability of Google Trends for studies other than infodemiological ones.”

8. Dear Reviewer, we again agree and thank you. We rewrote the section specifying the procedure in detail and why each source was chosen for analysis. Thank you very much for raising this essential inconsistency.

9. Dear Reviewer, this paper aims not only to understand if there is an influence of the media on web searches but also to quantify it. The correlational analysis shows strong evidence in favor of a marked and statistically significant influence both in the adoption of keywords and monikers and search trends. These results can help understand the overriding need to provide quality information (especially during health emergencies) and the relevance of the media in the diffusion of the latter.

10. Dear Reviewer, thank you for noting this. The following explanation was provided in the manuscript: “We exploited the infodemic scale (I-scale) to assess the infodemicity of the terms examined: each moniker was assigned 1 to 2 points per category (ie, generic, misinformative, discriminatory, deviant, other specificities), ranging from 0 to 10. Based on the sum of the I-scale scores, the infodemic monikers were classified as follows: not infodemic (0), slightly infodemic (1), moderately infodemic (2-4), highly infodemic (5-8), and extremely infodemic (9-10). Further details on the use of the I-scale are given in reference 10.”

11. Dear Reviewer, we agree with the difference you highlighted between the types of data. In this regard, we considered that the correlational analysis (unlike the regression) is not influenced by absolute values but only by trends, which are not affected by normalization. This paper relates relative web interest (relative search volume) to newspaper headlines but not the exact number of web searches. This was specified in the *Limitations* section. However, since it is known from other papers that the number of searches on COVID-19 has always been high [4], even relative trends provide valuable information. Similar analyzes have been made, for example, between RSV and COVID-19 cases (eg, [5]).

12. Dear Reviewer, thank you for the question. As we have now added in the paper, “To evaluate time-series seasonalities, we used a graphical check and the ‘Time Series Analysis’ tool of the XLSTAT package. In particular, we have divided the signal into the trend, seasonal, and random components. Finally, we calculated the autocorrelogram plot.”

13. Dear Reviewer, we did as requested. Thanks.

14. Dear Reviewer, thank you very much for raising this interesting point. The *Discussion* section has been rewritten to include these topics.

15. Thank you so much for the valuable comment. The *Discussion* section has been redesigned and divided into the following parts: *Principal Findings*, *Comparison With Recent Literature*, *Practical Implications*, *Limitations*, and *Conclusions*.

16. Once again, thank you so much for highlighting this essential aspect. The new *Discussion* section addresses these points based on what is found in this paper and on previous literature.

17. Done. We thank you very much for your help in improving our manuscript.

### Anonymous

1. Dear Reviewer [6], we appreciate your constructive criticism. The *Introduction* section has been rewritten and enriched following your suggestions. Thank you very much.

2. Dear Reviewer, we fully agree with your comment and thank you. The *Data Collection* section is now explained in more detail. Furthermore, we have added Google Trends among the sources, specifying the geographic region (Italy, national data), the type of data analyzed (web searches), and the category (all categories). Keywords and search periods are shown in Table 1. In addition, we have removed unnecessary information from the statistical analysis as required (we have just left the legend).

3. Dear Reviewer, thank you very much for your valuable suggestion. We have significantly lightened the *Results* section by including more data in the supplementary file and removing redundant information. Furthermore, we simplified the correlational analysis by introducing a cross-correlation summary result.

4. Dear Reviewer, the section has been completely rewritten. Thank you for raising this relevant issue.

5. Dear Reviewer, thanks for the helpful information. We have arranged the Abstract as requested.

6. Dear Reviewer, we have removed the vast majority of bullet points. Thanks for your help.

7. Dear Reviewer, we did as requested. We thank you very much for your help in improving our paper.
